# Extreme weather caused by concurrent cyclone, front and thunderstorm occurrences

**DOI:** 10.1038/srep40359

**Published:** 2017-01-11

**Authors:** Andrew J. Dowdy, Jennifer L. Catto

**Affiliations:** 1 Bureau of Meteorology, Docklands, 3007, Australia; 2 Monash University, Clayton, 3800, Australia

## Abstract

Phenomena such as cyclones, fronts and thunderstorms can cause extreme weather in various regions throughout the world. Although these phenomena have been examined in numerous studies, they have not all been systematically examined in combination with each other, including in relation to extreme precipitation and extreme winds throughout the world. Consequently, the combined influence of these phenomena represents a substantial gap in the current understanding of the causes of extreme weather events. Here we present a systematic analysis of cyclones, fronts and thunderstorms in combination with each other, as represented by seven different types of storm combinations. Our results highlight the storm combinations that most frequently cause extreme weather in various regions of the world. The highest risk of extreme precipitation and extreme wind speeds is found to be associated with a triple storm type characterized by concurrent cyclone, front and thunderstorm occurrences. Our findings reveal new insight on the relationships between cyclones, fronts and thunderstorms and clearly demonstrate the importance of concurrent phenomena in causing extreme weather.

Recent years have seen a growing interest in the socioeconomic and biophysical impacts of extreme weather events^1^,^2^,^3^,^4^,^5^,^6^,^7^,^8^,^9^ including how they relate to climate change^10^,^11^,^12^,^13^,^14^,^15^,^16^ and sustainable development^17^,^18^,^19^,^20^ throughout the world. However, a current knowledge gap in understanding the causes of extreme weather events is the role of concurrent phenomena, noting that different types of phenomena such as cyclones, fronts and thunderstorms can sometimes occur simultaneously in the same geographic region (i.e., concurrently)^5^,^21^,^22^,^23^,^24^. Consequently, an improved understanding of extreme weather caused by concurrent phenomena could have important implications for a range of fields including disaster risk reduction^20^, climate adaptation^4^, emergency management^25^,^26^, insurance^16^,^27^, economics^18^, agriculture^28^, ecology^14^,^29^,^30^ and health^17^,^26^,^31^.

Extreme events can be defined at a number of different temporal scales, including multi-year return periods based on statistical inferences^32^, as well as percentile measures based on events that occur within a given time period^33^. A number of recent studies^3^,^5^,^8^,^34^,^35^,^36^,^37^,^38^ have applied broad-scale systematic methods to examine the characteristics of percentile-based extreme weather events at the spatial and temporal scales provided by global reanalysis datasets, with this type of approach being similar to that applied here.

Here we examine different combinations of cyclones, fronts and thunderstorms, as represented by seven different types of storm combinations: Cyclone Only (CO), Front Only (FO), Thunderstorm Only (TO), Cyclone and Front (CF), Cyclone and Thunderstorm (CT), Front and Thunderstorm (FT) and Cyclone, Front and Thunderstorm (CFT). Systematic detection algorithms are applied to ERA-Interim reanalyses^39^ to examine the occurrence of fronts^40^,^41^,^42^ and cyclones (including extratropical and tropical systems)^3^,^43^, with the cyclone occurrences supplemented by the addition of global tropical cyclone data^44^. Thunderstorm occurrences are based on lightning observations from a global network of ground-based sensors^45^,^46^, with data available for complete years from 2005 onwards defining the start of the time period considered in this study. The systematic approach applied here based on combining cyclone, front and thunderstorm occurrence data aims to address knowledge gaps associated with the causes of extreme precipitation events and extreme wind events, so as to help lead to improved resilience to the impacts that these events can have throughout the world.

## Results

### Storm combinations and associated extreme weather

**Figure 1:**
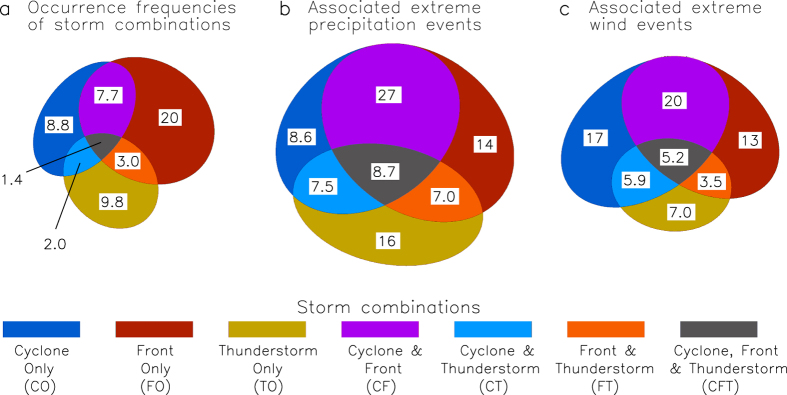
Storm combinations and associated extreme weather. Occurrence frequencies of the seven different storm combinations are shown (**a**), as well as the proportion of extreme precipitation events associated with each storm combination (**b**) and the proportion of extreme wind events associated with each storm combination (**c**). The values shown (in %) represent the mean for the entire study region (70°N–70°S in latitude, globally in longitude) and time period (2005–2015), with the size of the coloured area shown for each storm combination being proportional to these values.

Figure 1 presents the different combinations of cyclone, front and thunderstorm occurrences, as represented by the seven different types of storm combinations considered throughout this study (i.e., CO, FO, TO, CF, CT, FT and CFT). Occurrence frequencies for each of the storm combinations are shown, as well as how often they occur at the same location as an extreme precipitation or wind speed event. All analyses are based on 6-hourly values during the period 2005–2015, using a spatial grid of 0.75° throughout the region 70°N–70°S in latitude and globally in longitude. Precipitation and wind speed (wind gust at 10 m) are obtained from ERA-Interim reanalysis^39^, with extreme values defined here as being greater than the 99^th^ percentile (calculated individually for each grid-cell using all available times). Further details on data and methods are provided in the Methods section.

The triple storm type (i.e., CFT) is the rarest of the seven different storm combinations (Fig. 1a), with the double storm types also occurring relatively infrequently (7.7% for CF, 2.0% for CT and 3.0% for FT) as compared to the single storm types (8.8% for CO, 20% for FO and 9.8% for TO). However, even given the scarcity of the triple and double storms, these concurrent storm types collectively account for 50% of all extreme precipitation events (Fig. 1b) and 35% of all extreme wind events (Fig. 1c), highlighting the importance of the combined influence of different phenomena in causing extreme weather events.

Of the seven different types of storm combinations, the highest risk of an extreme precipitation event occurring is associated with the triple storm type (CFT), given that they account for 8.7% of all extreme precipitation events despite occurring only 1.4% of the time. Similarly, the highest risk of an extreme wind event occurring is also associated with the triple storm type (CFT), accounting for 5.2% of all extreme winds events.

### Variation with latitude

**Figure 2:**
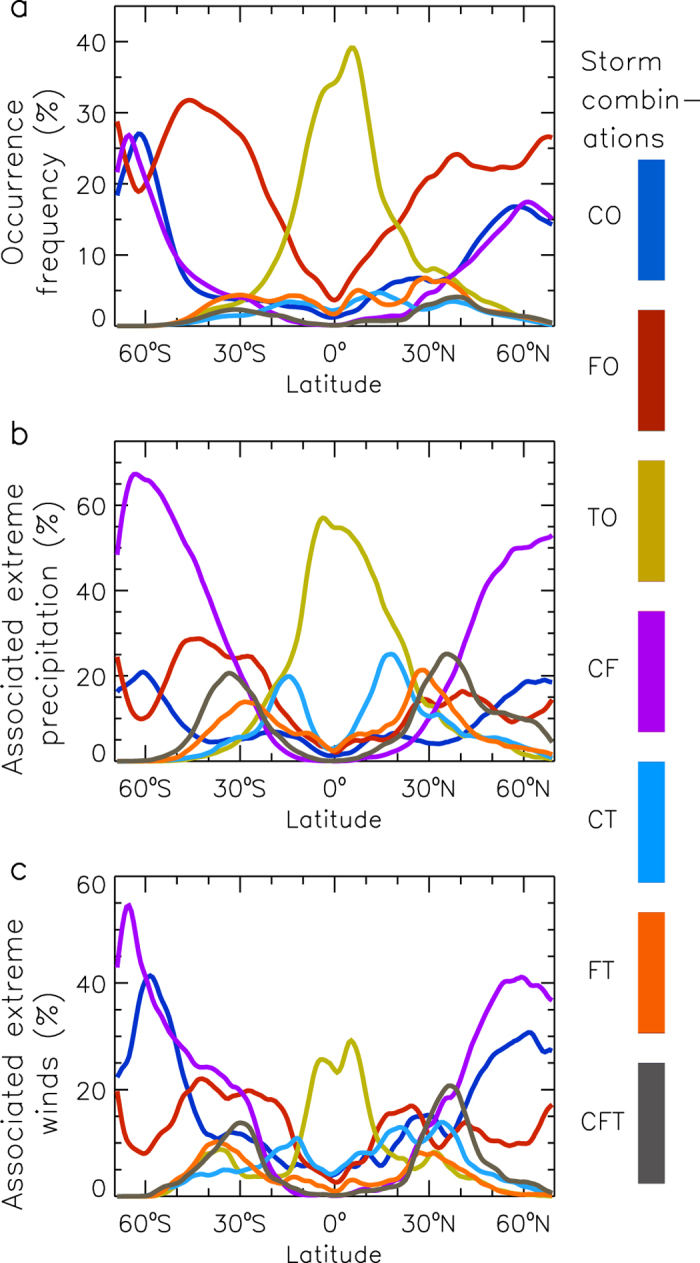
Variation with latitude of the storm combinations and associated extreme weather. Mean values are shown for different latitudes within the study region (70°N–70°S in latitude, globally in longitude), based on the time period 2005–2015. Results are shown for the occurrence frequency of each storm combination (**a**), the proportion of extreme precipitation events associated with each storm combination (**b**) and the proportion of extreme wind events associated with each storm combination (**c**).

There is considerable variation with latitude in the occurrence frequencies of the storm combinations and their associated extreme weather events, as shown by the zonal-mean values presented in Fig. 2. The most common type of storm combination in the tropics is TO, while at higher latitudes the most common type is FO with the exception of the region around 60°S (near the Southern Hemisphere storm tracks around Antarctica) where the CO and CF types frequently occur (Fig. 2a).

Extreme precipitation (Fig. 2b) and wind speeds (Fig. 2c) are most frequently associated with the TO type in the tropics. In midlatitude regions, extreme precipitation and wind speeds are frequently caused by a wide range of different types (including CT, FT and CFT), despite the fact that the FO type occurs more frequently than any other storm type at these latitudes (Fig. 2a). At higher latitudes, the extreme weather events are most frequently associated with the CF type, particularly in the Southern Hemisphere, noting that extreme winds in the region 50°S–60°S are also frequently associated with the CO type.

### Environmental characteristics

**Figure 3:**
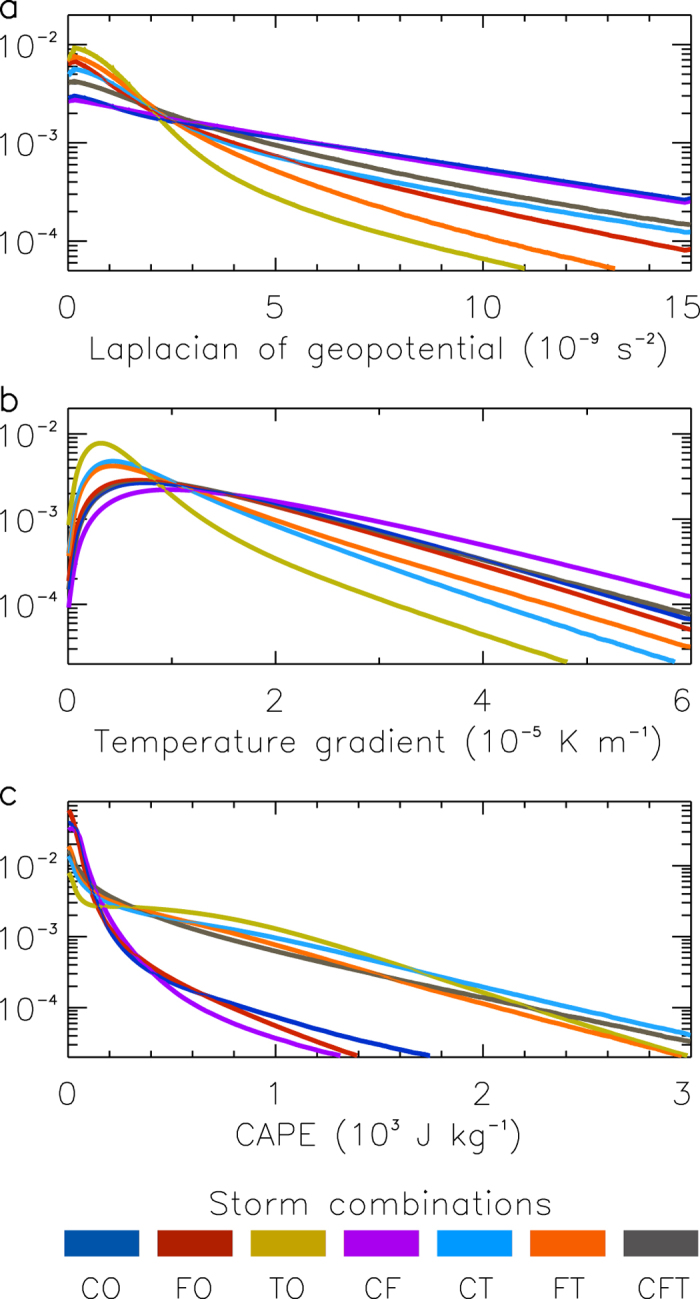
Environmental characteristics associated with the storm combinations. Normalised frequency distributions are shown for three different atmospheric measures: Laplacian of geopotential at 500 hPa (**a**); gradient of the temperature field at 700 hPa (**b**); and convective available potential energy (CAPE) (**c**). The distributions are calculated individually for each of the storm combinations throughout the study region (70°N–70°S in latitude, globally in longitude) and time period (2005–2015).

Figure 3 examines the characteristics of the storm combinations as represented by frequency distributions of three atmospheric measures (based on ERA-Interim reanalyses^39^ as detailed in the Methods section): the Laplacian of geopotential at the 500 hPa pressure level (LapG500), the magnitude of the temperature gradient at the 700 hPa pressure level (GradT700) and convective available potential energy based on near-surface air parcels (CAPE). These atmospheric measures represent conditions that can indicate the potential occurrence of the three phenomena (i.e., LapG500 for cyclones, GradT700 for fronts and CAPE for thunderstorms) and are used here to examine the environmental conditions in the vicinity of cyclones, fronts and thunderstorms as represented by the seven different storm combinations.

The frequency distributions of the three atmospheric measures show clear variations between the different storm combinations, including for the concurrent storm types (i.e., the double and triple storm types). The upper tails of the distributions show that the double storm types are each associated with relatively high values of two of the three atmospheric measures (i.e., LapG500 and GradT700 for CF, LapG500 and CAPE for CT, as well as GradT700 and CAPE for FT). The triple storm (CFT) is the only type associated with relatively high values of all three measures. These results provide a plausible physical explanation for the triple storm type having a high risk of causing extreme weather, given that these three atmospheric conditions (in addition to those used to define this storm type) represent a combination of factors that can lead to severe weather conditions including low-level and high-level dynamic forcing, deep thermodynamic instability and potential triggers to initiate vertical motion (as can sometimes occur near frontal temperature gradients^41^,^47^).

### Geographic variations

**Figure 4:**
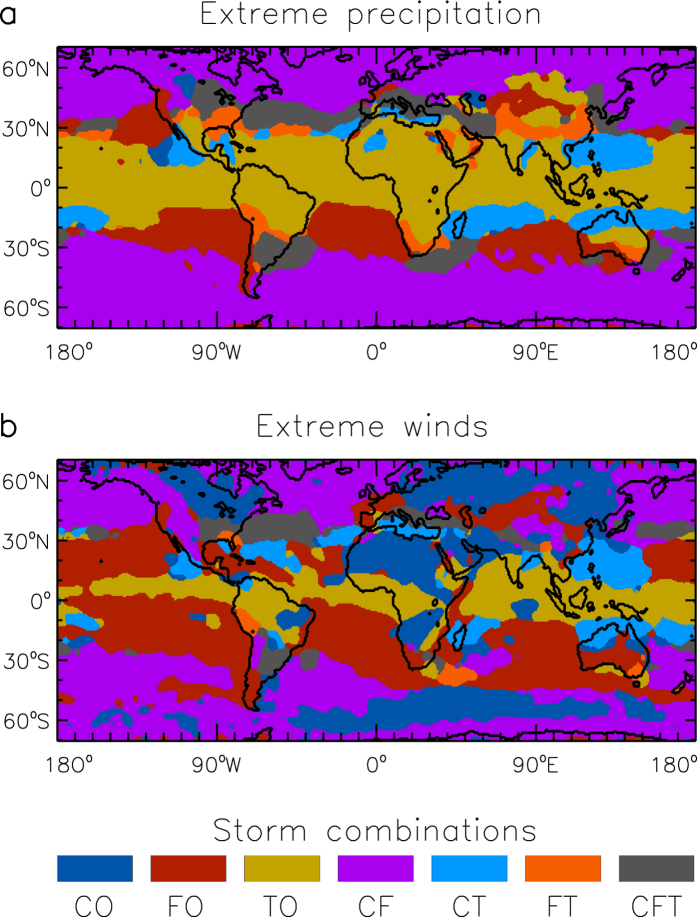
Common causes of extreme weather events during the time period 2005–2015. Maps are shown of the type of storm combination that is most frequently associated with extreme precipitation events (**a**) and extreme wind events (**b**). The land-sea mask of the ERA-Interim reanalysis is used for the coastlines shown here (data visualisations produced using IDL [8.5] (Exelis Visual Information Solutions, Boulder, Colorado)).

Figure 4 presents maps of the storm combinations that are most frequently associated with extreme precipitation (Fig. 4a) and extreme winds (Fig. 4b) at each individual location. There are clear regional features apparent from these maps. In particular, the results indicate that the triple storm type (CFT) is a common cause of extreme weather events in some midlatitude regions, with a considerable degree of regularity near the east coast of continents throughout the world. This suggests that triple storm events could have relevance in these regions to various coastal processes (e.g., erosion, storm surge, large waves and inundation) and activities (e.g., shipping, coastal zone management, renewable energy generation and recreational pursuits such as surfing and sailing). The triple storm events are also a common cause of extreme weather in some inland regions, including parts of North America, southern Europe, western Asia, China, Japan and Argentina (Fig. 4).

## Discussion

A greater understanding of extreme events represents a research priority of global importance^1^,^4^,^17^,^18^,^19^,^20^,^48^, noting that the physical characteristics and impacts of an extreme event (as well as other factors such as counter-response measures and forecast lead times) are in part dependent on the type of phenomenon, or combination of phenomena, that cause the extreme event to occur. Our results represent a significant advance in understanding the characteristics of extreme weather events, including in relation to concurrent phenomena, with the importance of the combined influence of cyclones, fronts and thunderstorms being clearly evident.

In addition to the results presented here, we foresee that the method applied in this study could be adapted for use in various future applications, including based on other data types (e.g., coarser-scale global climate model output or finer-scale observations for specific regions), other natural hazards (e.g., extreme ocean waves, temperatures or wildfire activity) and other phenomena (e.g., blocking^36^, jet structures^8^ or warm conveyor belts^37^,^38^). An improved ability to decompose different causes of extreme weather could also have benefits for climate modelling applications, such as for distinguishing different drivers of variability and constraining uncertainty estimates in projected changes to extreme events^11^,^15^,^16^,^48^,^49^,^50^,^51^, noting that many of the repercussions of a warmer world are expected to be experienced through changes to extreme weather events and associated natural hazards^9^,^12^,^13^,^14^,^25^,^26^,^27^,^30^.

The characteristics of phenomena such as cyclones, fronts and thunderstorms can vary over a wide range of temporal scales including potential long-term trends in their climatology, as well as shorter-term diurnal, seasonal and interannual variations. For example, variations in these phenomena can sometimes be associated with large-scale atmospheric and oceanic modes of variability such as the El Nino/Southern Oscillation (ENSO)^52^,^53^,^54^, suggesting that there might be potential for developing improved methods for predicting extreme weather events at seasonal time scales based on this method of considering combined storm types. Further scope for examining temporal variations in extreme weather events includes examining how global warming might influence the occurrence of the new type of storm identified here – the triple storm.

Our results provide new insight on the relationships between cyclones, fronts and thunderstorms, including with respect to the extreme precipitation and wind events that concurrent phenomena can cause in various regions of the world. It is intended that an improved understanding of extreme weather events will help lead to improved resilience to their impacts as well as inform the prioritisation of disaster risk reduction and adaptation efforts throughout the world^1^,^4^,^17^,^18^,^19^,^20^,^25^,^26^,^27^,^28^,^29^,^30^,^31^.

## Methods

### Identification of cyclones, fronts and thunderstorms

Cyclones (including tropical and extratropical systems) are identified here by applying a systematic detection method using mean sea level pressure (MSLP), as detailed in previous studies^3^,^5^,^43^. The method identifies MSLP minima based on the criterion that a region must have pressure lower than the surrounding grid-cells (using a contour interval of 0.5 hPa), with the region enclosed by the outermost closed contour considered as being within a cyclone. Consequently, this method is based on closed regions of MSLP contours while noting that there is a wide range of methods, including the method used here, that are frequently used to examine cyclones^7^,^51^. This method can be applied similarly in different regions of the world, including the tropics and the extratropics. It is applied to the global atmospheric reanalysis produced by the European Centre for Medium-Range Weather Forecasts (ECMWF), the ERA-Interim reanalyses product^39^, throughout the 11-year period 2005–2015 with a time step of 6-hours and a grid spacing of 0.75° in both latitude and longitude. As some tropical cyclones are not well-resolved at the scales of current reanalyses, the cyclone data are supplemented by the addition of global tropical cyclone data from the International Best Track Archive for Climate Stewardship (IBTrACS, v03r09^44^).

Fronts are identified here with an automated method^42^ using a thermal front parameter^40^,^41^, *TFP*, based on the 850 hPa wet bulb potential temperature, *θ*_*w*_, as shown in equation 1. The method firstly selects regions where *TFP* is less than a threshold value (−5 × 10^−11 ^K m^−2^), then secondly examines these regions for locations where the gradient of *TFP* is zero which are joined numerically into contiguous fronts, such that the front data include both cold and warm fronts. The method is applied here at all longitudes globally and from 70°N to 70°S in latitude, similar to previous applications of this method^5^,^34^,^42^. It is applied here to ERA-Interim reanalysis^39^, using the same gridded region and time period as used for the cyclone data. It is noted that there are a number of ways to identify fronts, each with their advantages and disadvantages^55^, with the thermal approach selected for use here due to its ability to identify both cold and warm fronts, given that both of these types of fronts can be associated with extreme weather events^5^,^38^.


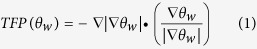


Thunderstorms are identified here based on lightning data obtained from a global network of ground-based sensors (the World Wide Lightning Location Network: WWLLN^45^,^46^). The lightning data are gridded on the same grid as used for the cyclone and front data (i.e., 6-hourly time steps and 0.75° grid spacing), noting that the WWLLN observations are available at finer spatial and temporal resolutions than are the focus of this study. Grid-cells containing thunderstorms are identified here based on two or more lightning strokes observed within the 0.75° × 0.75° region and 6-hour period represented by a particular grid-cell and time step, so as to provide an indication of a deep convective storm at a location within that region and time period. The lightning data are available for complete years from 2005 onwards, defining the start of the time period considered here.

### Extreme weather events

Extreme values of precipitation and wind speed for a given grid-cell are considered here as being greater than the 99^th^ percentile (calculated individually for each grid point location based on the entire period 2005–2015), noting that extremes can be defined at a number of different scales including more frequent events (e.g., based on the 90^th^ percentile^33^) or less frequent events (e.g., multi-year return periods^32^) than those considered here. Given the 6-hourly time steps and the 11-year period of available data, the use of the 99^th^ percentile to examine extreme events results in about 160 events at each grid-cell location, whereas the choice of a higher threshold value such as the 99.9^th^ percentile would result in too few events to produce robust findings.

Precipitation and wind speeds are obtained from ERA-Interim reanalysis^39^ using the same spatial and temporal grid as used for the front, cyclone and thunderstorm data (i.e., 6-hourly time steps and 0.75° grid spacing in both latitude and longitude). Although purely observational datasets are available they can be relatively limited in spatial extent, particularly in the case of wind data, such as for coverage in ocean regions and high latitudes in general. A benefit of using reanalyses for broad-scale systematic investigations is that they can provide global coverage based on regular spatial and temporal grids, noting that although some fine-scale aspects may not be well-represented in some cases (such as the magnitude of localised extremes associated with small-scale convective or microphysical processes) the method applied here only considers whether or not an extreme weather event occurs (i.e., avoids uncertainties associated with quantifying the degree of severity of an extreme event). Here we use the 6-hour forecast values of total precipitation and with wind speeds based on the parameterised wind gust at a height of 10 m, similar to previous studies^3^,^5^,^8^,^34^,^36^,^37^ that have examined percentile-based extreme weather events using ERA-Interim reanalysis^39^.

### Combining the cyclone, front and thunderstorm data

Phenomena such as cyclones, fronts and thunderstorms can influence weather conditions in their surrounding regions (e.g., as shown in Supplementary Fig. 1). Consequently, a ±3 grid-cell range of influence is applied to the raw data of these phenomena for use throughout this study (i.e., ±2.25° in both latitude and longitude, broadly similar to previous studies^2^,^5^,^34^), including for use in defining the different types of storm combinations considered in this study. The resultant occurrence data for the three phenomena are presented in Supplementary Fig. 2, as well as how often extreme weather events occur at the same location as these phenomena, showing similar features to previous studies^2^,^3^,^5^,^23^,^34^,^46^ noting some variation in methods and time periods between studies. The seven different types of storm combinations considered here are based on the different combinations of the cyclone, front and thunderstorm occurrence data at a given grid-cell and time step: Cyclone Only (CO), Front Only (FO), Thunderstorm Only (TO), Cyclone and Front (CF), Cyclone and Thunderstorm (CT), Front and Thunderstorm (FT) and Cyclone, Front and Thunderstorm (CFT). The area-proportional Euler diagrams in Fig. 1 provide general schematic representations of the storm combinations based on combining the cyclone, front and thunderstorm occurrence data.

### Atmospheric measures

Three atmospheric measures are used in this study to examine environmental characteristics in the vicinity of the seven different storm combinations. The atmospheric measures are the Laplacian of the geopotential field at the 500 hPa pressure level (LapG500), the vector gradient of the temperature field at the 700 hPa pressure level (GradT700) and convective available potential energy based on lifting near-surface air parcels (CAPE) as provided in the ERA-Interim reanalysis^39^. Normalised frequency distributions of these atmospheric measures are calculated for each of the seven storm combinations. The distributions are produced by counting the number of times that a given value of an atmospheric measure occurs, based on all instances of a particular storm combination throughout the study period and region, with this done for 1,000 segments (i.e., bins) of the atmospheric measure’s range (as shown in Fig. 3). The distributions are normalised by dividing by the sample size in each case.

## Additional Information

**How to cite this article**: Dowdy, A. J. and Catto, J. L. Extreme weather caused by concurrent cyclone, front and thunderstorm occurrences. *Sci. Rep.* **7**, 40359; doi: 10.1038/srep40359 (2017).

**Publisher's note:** Springer Nature remains neutral with regard to jurisdictional claims in published maps and institutional affiliations.

## Supplementary Material

Supplementary Information
