# Supplementary material for: Extreme weather caused by concurrent cyclone, front and thunderstorm occurrences
Source: Sci Rep. 2017 Jan 11;7:40359. doi: 10.1038/srep40359 (PMC5225482; doi:10.1038/srep40359)
Supplement: Supplementary Information [file srep40359-s1.pdf]

Supplementary information:

# Extreme weather caused by concurrent cyclone, front and thunderstorm occurrences

**Andrew J. Dowdy[1,*], Jennifer L. Catto[2]**

[1]Bureau of Meteorology, Docklands, 3007, Australia

[2]Monash University, Clayton, 3800, Australia

[*]a.dowdy@bom.gov.au

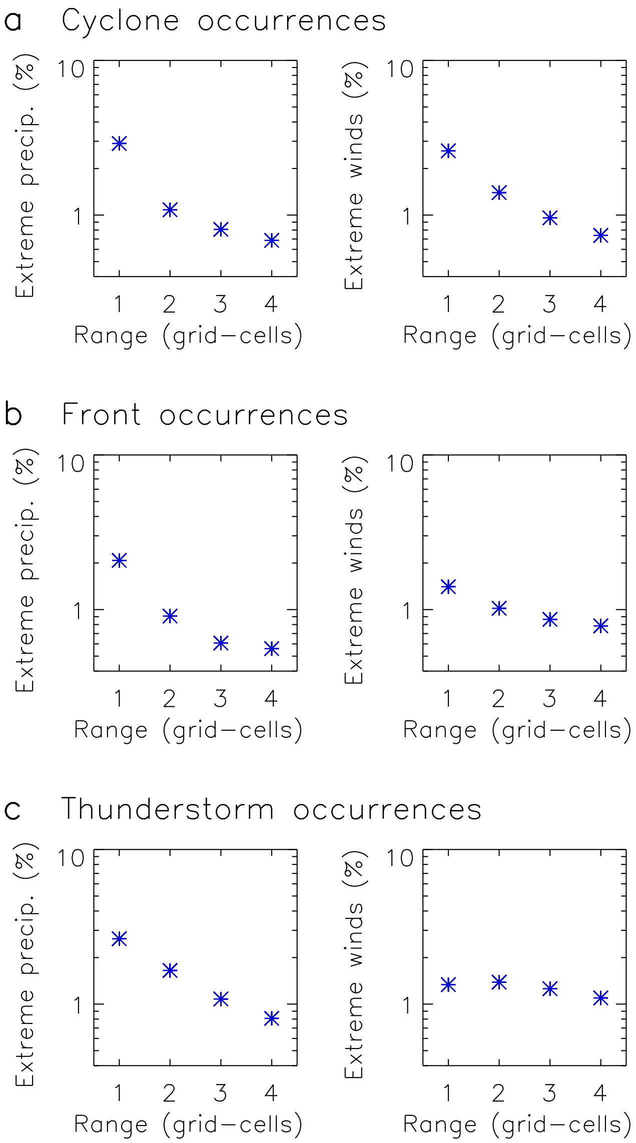


**Supplementary Figure 1.** Spatial influence of the three phenomena on extreme weather events. The percentage of locations that have an extreme weather event is shown at different ranges from the raw data for the three phenomena: cyclones (a); fronts (b); and thunderstorms (c). For each time step, individual grid-cells are only counted once (for each of the six cases shown) at the smallest range from that location to the particular phenomenon being considered. Mean values are shown for the entire study region and time period, for extreme precipitation events (left images) and extreme wind events (right images).

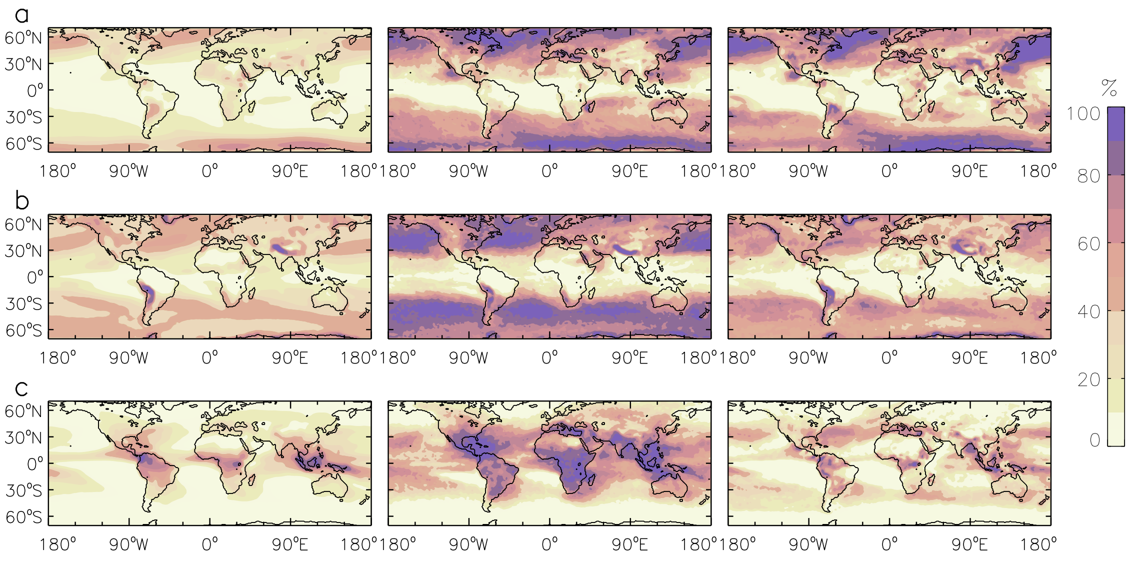


**Supplementary Figure 2.** Frequency of occurrence of the phenomena and associated extreme weather. Mean values are shown for the time period 2005–2015 for cyclones (a), fronts (b) and thunderstorms (c). The occurrence frequency of these phenomena is shown (left column images), as well as how often extreme precipitation events (middle column images) and extreme wind events (right column images) occur in association with these phenomena. The land-sea mask of the ERA-Interim reanalysis is used for the coastlines shown here (data visualisations produced using IDL [8.5] (Exelis Visual Information Solutions, Boulder, Colorado)).
